# Clinical services for adults with an intellectual disability and epilepsy: A comparison of management alternatives

**DOI:** 10.1371/journal.pone.0180266

**Published:** 2017-07-03

**Authors:** Adam P. Wagner, Tim J. Croudace, Naomi Bateman, Mark W. Pennington, Elizabeth Prince, Marcus Redley, Simon R. White, Howard Ring

**Affiliations:** 1 National Institute for Health Research Collaboration for Leadership in Applied Health Research and Care East of England, Cambridge, United Kingdom; 2 Norwich Medical School, University of East Anglia, Norwich, United Kingdom; 3 Department of Psychiatry, University of Cambridge, Cambridge, United Kingdom; 4 School of Nursing and Midwifery and Social Dimensions of Health Institute, University of Dundee, Dundee, United Kingdom; 5 King’s Health Economics, King’s College London, London, United Kingdom; 6 Medical Research Council Biostatistics Unit, University of Cambridge, Cambridge, United Kingdom; 7 Cambridgeshire and Peterborough National Health Service Foundation Trust, Cambridge, United Kingdom; TNO, NETHERLANDS

## Abstract

**Background:**

Intellectual disability (ID) is relatively common in people with epilepsy, with prevalence estimated to be around 25%. Surprisingly, given this relatively high frequency, along with higher rates of refractory epilepsy than in those without ID, little is known about outcomes of different management approaches/clinical services treating epilepsy in adults with ID—we investigate this area.

**Materials & methods:**

We undertook a naturalistic observational cohort study measuring outcomes in n = 91 adults with ID over a 7-month period (recruited within the period March 2008 to April 2010). Participants were receiving treatment for refractory epilepsy (primarily) in one of two clinical service settings: community ID teams (CIDTs) or hospital Neurology services.

**Results:**

The pattern of comorbidities appeared important in predicting clinical service, with Neurologists managing the epilepsy of relatively more of those with neurological comorbidities whilst CIDTs managed the epilepsy of relatively more of those with psychiatric comorbidities. Epilepsy-related outcomes, as measured by the Glasgow Epilepsy Outcome Scale 35 (GEOS-35) and the Epilepsy and Learning Disabilities Quality of Life Scale (ELDQoL) did not differ significantly between Neurology services and CIDTs.

**Discussion:**

In the context of this study, the absence of evidence for differences in epilepsy-related outcomes amongst adults with ID and refractory epilepsy between mainstream neurology and specialist ID clinical services is considered. Determining the selection of the service managing the epilepsy of adults with an ID on the basis of the skill sets also required to treat associated comorbidities may hence be a reasonable heuristic.

## Introduction

Epilepsy is common in adults with intellectual disability (ID), as the most frequent serious medical illness experienced by this clinical group [1], with an overall prevalence of around 26% [2]. Epilepsy in adults with ID has a worse prognosis than epilepsy in the general population, with lower rates of seizure freedom [3] and high rates of multiple anti-epileptic drug use [4], which incur more side-effects [5]. Adults with ID and epilepsy also have high rates of morbidity [6] and mortality, including sudden unexplained death in epilepsy [7]. Poorly controlled and inadequately treated epilepsy also impacts negatively on the quality of life of adults with ID and epilepsy, and associated consequences affect families and paid carers [8–9].

Surprisingly, given these poor outcomes, little is known about the best approach to epilepsy management for this population and guidelines are largely based on anecdote or clinical consensus [10]. Among those with epilepsy, people with ID have a greater prevalence of complex comorbidities to deal with relative to those without ID [11–12]. These comorbidities can often interact with their epilepsy, making their care and treatment even more challenging. This has resulted in some regions having a wide range of healthcare providers delivering aspects of epilepsy-related care to adults with ID (ranging from primary care, to specialist community health services for people with ID, to Neurology services). Neurology services tend to be based in secondary or tertiary care health care settings, with a focus on treating neurological disease; whilst, in the UK, multidisciplinary health teams for people with ID (CIDTs) are based in the community and generally have a wider remit, managing a range of psychobehavioural morbidities and functional difficulties. Given this choice of potential healthcare providers it is important to consider whether there are differences between providers in terms of the patients they treat and the outcomes they obtain. Previous research in one region of England has demonstrated that most adults with ID and epilepsy receive their epilepsy management from community-based ID services or from hospital-based Neurology services, whilst a smaller proportion receive epilepsy management from multiple sources including General Practitioners [4]. This current disposition of epilepsy services for adults with ID and epilepsy in the UK provides a natural situation in which to investigate the type of person using the different types of service and whether individuals fare better in one type of service than in another.

Therefore, using an observational cohort methodology, we have undertaken research to test the hypothesis that amongst 91 adults with ID and epilepsy, recruited from community ID teams (CIDTs) or hospital Neurology services, there are differences between the recipients of these two services. The results from this study may help those who commission and design services as well as clinicians to determine which service is best suited to supporting a particular individual’s epilepsy.

## Materials & methods

### Participant recruitment, inclusion criteria and sample size

Recruitment and data collection took place between March 2008 and April 2010. Eligible participants comprised all adults aged between 18 and 65 years with epilepsy and at least one seizure in the preceding 6 months, with a diagnosis of a full scale IQ below 71, living in Cambridgeshire or Norfolk and known to community ID and/or hospital Neurology services. Potential participants were initially identified by the clinicians managing their epilepsy. All potential participants identified (334) were contacted and 198 replied. Of these, 28 were ineligible and in 79 cases consent or agreement from a consultee was not given, leaving 91 individuals to participate in the study (who provided informed written consent, or for whom a written favourable opinion from a consultee was given, as appropriate). For those lacking capacity to consent, advice was sought from a carer under the provisions of the Mental Capacity Act [13]. Forty six (51%) of the 91 participants were male. The mean age of participants was 41.3 years (standard deviation, SD = 11.8) and the mean duration of epilepsy was 30.7 years (SD = 16.1, in the 79 participants providing this data). The study was approved by the Cambridgeshire 2 Research Ethics Committee.

### Study design and data collection

Following recruitment, each participant was visited five times over a period of seven months in order to obtain repeated measures of their clinical state, which could be averaged over a period of time. This aims to reduce the effect on these observations of the short-term variability in seizure frequency that occurs in a proportion of those with epilepsy.

During the first visits to participants’ homes, consent or favourable advice was obtained and background information was collected describing the participant’s epilepsy and its treatment, ID severity and comorbid medical conditions, along with details of their accommodation. The subsequent visits occurred at around one, two, six and seven months after the initial visit. During these four subsequent assessment visits, qualitative information, health economic data and outcome measures, including the Glasgow Epilepsy Outcome Scale 35 (GEOS-35) and the Epilepsy and Learning Disabilities Quality of Life Scale (ELDQoL), were collected. In this paper we restrict our attention to clinical outcomes described by the GEOS-35 and the ELDQoL. Analysis from other measures has been reported elsewhere [14–15].

### Measures

#### GEOS-35

The GEOS is a carer-reported measure of outcomes in epilepsy developed for use with people who have ID. The GEOS-35 is a short form of the GEOS-90 [16], comprising four sub-scales recording the informant’s concerns about: Seizures (10 items); Medical treatment (9); Caring for a person with epilepsy (8); and Social impact of epilepsy (8). GEOS items require responses that are rated on a five point scale, from “0 –never a concern/not applicable” to “4 –very often a concern”. A higher score on any of the sub-scales, or the overall score, indicates a greater level of concern. Espie et al. [17] show that both the GEOS-90 and GEOS-35 have good internal consistency (Cronbach’s alpha>0.8), and acceptable discriminant and concurrent validity.

We aimed to complete the GEOS-35 on the first and fourth assessment visits. The measure was either completed by a family carer or paid support worker. Differences in concerns reported by different types of carer have been investigated: high inter-rater agreement was found between family members but there was considerable variability between support workers [18]. Thus, when support workers completed this form, we sought to have the same individual complete the measure at both collection points. In the following analyses, for each sub-scale and for the overall score, we use the mean of scores from assessment one and assessment four. For seven participants the GEOS-35 was completed only once.

#### ELDQoL

The ELDQoL is a 70 item carer-reported measure examining seizure severity, seizure related injury, Anti-Epileptic Drugs (AED) side effects, behaviour, mood, physical status, cognitive and social functioning, communication, overall health and quality of life, and family concerns. Particular items in the Seizure severity (14 items), Side effects (19), Behaviour (9) and Mood (16) domains can be summed to create four sub-scales. Higher scores indicate a poorer quality of life. Buck et al. [19] explore the ELDQoL psychometric properties for collecting information about epilepsy in children and find that the ELDQoL has: good internal consistency (Cronbach’s alpha: 0.74–0.95); high reliability (range: 0.8–0.96); and good construct validity. While not validated for use with adult participants, other studies have used it with this group [20–22].

The ELDQoL was completed at each of the four assessment visits, gathering information about the participant’s epilepsy over the preceding four weeks. The Seizure severity sub-scale was completed separately for each type of seizure experienced by a participant, up to a maximum of five seizure types. Overall seizure severity was quantified for each participant as the mean of mean scores for each seizure type, providing an indication of the average severity of their epilepsy over that period.

### Dealing with missing data

Where a participant’s score on a sub-scale of a measure was affected by items with missing data, the missing values were replaced with an average of the items with complete data. This imputation only took place if more than 50% of items on any sub-scale had complete data; where more than 50% of the items on a scale were missing data, the participant was recorded as having a missing value for that particular sub-scale. Otherwise, no methods were used to correct for missing data; however, levels of missing data were generally low (particularly on the outcome measures).

### Analysis

We deal with the following types of data in this paper: first, ‘casemix’ variables (variables that describe the following aspects of each participant: ID severity; accommodation type; total number of AEDs taken by each participant; whether or not an Epilepsy Nurse Specialist has been seen in the preceding year; the presence of current comorbid neurological diagnoses; the presence of current comorbid psychiatric diagnoses; and the number of seizure types); secondly, we consider their clinical service (comprising CIDT, Neurology and ‘Other’); and thirdly, their scores on the outcome scales (GEOS-35 and ELDQoL). We proceed by identifying the primary clinical service supporting individuals by using latent class analysis (LCA). Subsequently, we use logistic regression to compare the casemix profiles between the CIDT and Neurology services. Wilcoxon tests were used to compare GEOS-35 and ELDQoL outcomes between CIDT and Neurology services.

## Results

### Identification of the treating clinical service

There was a lack of clarity in the identification of which service was responsible for epilepsy management for some participants, as in some instances there were differing opinions between the participant’s main carer, GP, ID Psychiatrist (if involved) and Neurologist (if involved). There were sixteen cases where there was disagreement between two or more of the opinions: seven cases went on to be identified as having their epilepsy managed by CIDT, four by Neurology and five fell into the ‘Other’ category. In fifteen of the sixteen cases, there was disagreement between carer and GP opinions. The corresponding patients had the full range of LD severity (mild—two; moderate—four; severe—six; profound—three), and mostly lived in group homes or supported living (nine) or the family home (six), with one patient living independently.

Therefore, to allocate participants to a clinical service, we used a model-based approach based on LCA. Latent class modelling has similar goals to cluster analysis (grouping individuals) but is based on a statistical model, not distance metrics. Our modelling used LCA to compare the fit of models with 2, 3 or 4 latent classes. The data supplied to the model were the opinions as to the managing clinician from the participant’s main carer, GP, LD Psychiatrist (if indicated) and Neurologist (if indicated) [23]. The model tries to find homogeneous groups within which, once we know their group membership (latent classification) there is no further association among the observed data (their responses). In this way, as we increased the number of classes, the LCA found a model that allocated participants to one of three groups used in our analyses [24]. Utilising LCA in this way allowed us to resolve disagreements in a pragmatic fashion that required no initial assumptions from the research team about whose opinions should take priority; otherwise, we might have to make subjective assumptions (eg in case of disagreement, assume the opinion of the GP is correct) or exclude those cases where there is disagreement.

Utilising LCA, forty-two participants were identified as having their epilepsy care managed by a CIDT and 37 as having it managed by a Neurology service. The `Other’ group consisted of 11 participants where epilepsy management was either provided only by the GP or by a combination of services, with no clear indication of which service led treatment. One participant was not assigned to any of these groups. The identity of the managing clinical service, as reported by the informants, did not change for any of the participants during the study.

### Profile of casemix variables within each clinical service

Table 1 shows the profiles of casemix variables within each of the clinical services. Contrasting the Other group with CIDT and Neurology is difficult since it contains few study participants; however, Table 1 suggests that the Other group is more likely to contain people with severe or profound ID, with a current comorbid neurological diagnosis, who are taking fewer anti-epileptic drugs. We do not consider the Other group further.

**Table 1:** The profile of patient heterogeneity (‘casemix’) variables within each of the clinical services and the Other group. CIDT = Community Intellectual Disability Team. ENS = Epilepsy Nurse Specialist.

Case mix variable		CIDT (N = 42)	Neurology (N = 37)	Other (N = 11)
Severity of intellectual disability	Mild	15%*	20%^†^	0%
	Moderate	20%*	31%^†^	18%
	Severe	49%*	31%^†^	45%
	Profound	17%*	17%^†^	36%
Accommodation	Group home/supported living	74%	49%	73%
	Family home	21%	41%	27%
	Independent	5%	11%	0%
Anti-epileptic drugs used	≥2	71%	81%	45%
ENS seen in last year	Yes	7%	46%	0%
Current comorbid neurological diagnosis	Present	10%	16%	36%
Current comorbid psychiatric diagnosis	Present	52%	32%	18%
Seizure types reported	1	48%	46%^†^	64%
	2	45%	34%^†^	27%
	≥3	7%	20%^†^	9%

*N = 41.

^†^N = 35. Percentages may not sum to 100 due to rounding.

Differences in casemix variables between CIDT and Neurology services were tested in R [25] using both unadjusted univariate comparisons (Fisher’s exact test and two sample t-tests) and adjusted comparisons utilising logistic regression. The results of the tests are given in Table 2.

**Table 2:** Unadjusted and adjusted comparisons in casemix variables (along with gender and age) between CIDT (reference level) and Neurology. **Unadjusted univariate comparisons made using Fisher’s exact test or two sample t-test (for age). Adjusted comparisons are estimated from a logistic model (with service type as response variable) including all the variables listed in the Table**. OR = odds ratio. ENS = epilepsy nurse specialist. diag = diagnosis.

Model term	Level	N	Unadjusted			Adjusted		
			OR/mean	95% CI		Odds ratio	95% CI	
				Lower	Upper		Lower	Upper
**Severity of intellectual disability**	Mild	12		-		-	-	-
	Moderate	18	1.24	0.23	6.85	8.90	0.65	210.95
	Severe	31	0.56	0.12	2.65	1.27	0.10	18.89
	Profound	13	0.86	0.14	5.36	0.48	0.02	10.85
**Accommodation**	Group home or supported living	45	-	-		-	-	-
	Family home	24	3.27*	1.06	10.71	50.89*	5.70	850.28
	Independent	5	2.93	0.30	38.63	46.68	0.99	4537.41
**Total number of anti-epileptics**	0 or 1	18	-	-		-	-	-
	2 or more	56	1.85	0.55	6.89	11.23*	1.54	137.83
**Seen an ENS during last year?**	No	57	-			-	-	-
	Yes	17	9.04*	2.16	54.97	55.58*	8.03	758.67
**Current comorbid neurological diag**.	No	64	-			-	-	-
	Yes	10	2.04	0.43	10.81	12.90*	1.60	146.91
**Current comorbid psychiatric diag**.	No	42	-			-	-	-
	Yes	32	0.48	0.16	1.35	0.06*	0.01	0.32
**(Max) Seizure types reported**	One	33	-	-		-	-	-
	Two	31	0.86	0.28	2.61	0.76	0.14	4.13
	≥ three	10	3.08	0.57	21.79	4.52	0.49	50.20
**Gender**	Male	35	-	-	-	-	-	-
	Female	39	0.92	0.33	2.54	0.77	0.17	3.43
**Age (years)**	CIDT **mean**	-	41.83	38.22	45.44	1.05	0.98	1.14
	Neurology **mean**	-	39.91	39.91	44.51			
**Intercept**	-	-	-	-	-	0.00*	0.00	0.36

‘*’ denotes significance at the 5% level. CI = Confidence Interval. R^2^ = .45 (Hosmer-Lemeshow), .46 (Cox-Snell), .61 (Nagelkerke). χ^2^(11) = 45.296, p<0.001

Confidence intervals for the odds ratios are wide given the small sample. Whilst the profile of ID severity between the clinical services did not differ significantly, it was included *a priori* as a proxy for features not captured by the casemix variables employed. We found that, compared to ID services, Neurology services were more likely to: support those living in a family home or independently, as opposed to residing in a group home or supported living environments; treat using more anti-epileptic drugs; support those with a current comorbid neurological diagnosis; and support those who had seen an Epilepsy Nurse Specialist in the preceding twelve months. Neurology services less frequently managed the epilepsy of those with a current comorbid psychiatric diagnosis. The direction of observed differences is the same in the unadjusted and adjusted comparisons, but the effects are much stronger in the adjusted comparisons.

### Differences in GEOS-35 and ELDQoL outcomes by clinical service

Table 3 shows who completed the GEOS-35 (completed up to two times) and the ELDQoL (completed up to four times). Within each measure, the majority of returns for each patient were completed by the same informant (top two rows of Table 3). A larger proportion of informants for CIDT were (paid) carers, while there was closer to an equal split between carers and family in Neurology. GEOS-35 and ELDQoL returns differ between themselves and the numbers given in Table 1 due to some informants not completing some measures.

**Table 3:** Details of who completed the GEOS-35 and ELDQoL measures, from which the scales used to compare the clinical services in Table 4 are formed.

Respondent type	GEOS-35 returns (completed up to 2 times)		ELDQoL returns (completed up to 4 times)	
	CIDT	Neurology	CIDT	Neurology
All returns completed by the same carer	27	14	25	14
All returns completed by the same family member	10	14	9	14
Returns completed by different carers	5	5	7	5
Returns completed by different family members	0	0	1	2
Returns completed by a mix of carers and family members	0	2	0	2

Table 4 shows the average outcome scales scores for each clinical service. Based on independent Wilcoxon tests, at the 5% level of significance, no significant differences in GEOS-35 or ELDQoL outcomes were found between those receiving their epilepsy management from a CIDT and those receiving it from a Neurology service.

**Table 4:** The profile of outcome scales variables within each of the clinical services and the Other group. GEOS-35 = Glasgow Epilepsy Outcome Scales-35. ELDQoL = Epilepsy and Learning Disabilities Quality of Life. CIDT = Community Intellectual Disability Team.

Scale		Scale details		Medians			Wilcoxon test of CIDT and Neurology scores
		Min	Max	CIDT (N = 41)	Neurology (N = 32)	Other (N = 10)	
**GEOS-35**	Seizures	0	40	9.5	8.0	6.1	W = 759, p = 0.8100
	Medical treatment	0	36	6.2	6.8	6.9	W = 716, p = 0.8499
	Caring	0	32	4.4	6.5	2.7	W = 595, p = 0.2155
	Social impact	0	32	2.9	5.9	2.4	W = 550, p = 0.1155
	GEOS-35 Total	0	140	23.8	30.3	17.5	W = 705, p = 0.7641
**ELDQoL**	Seizure severity (mean-avg)	10	56	25.6	24.6	23.7	W = 765, p = 0.7628
	Side effects	19	76	25.2	24.7	23.6	W = 712, p = 0.7907
	Behaviour	9	36	15.0	14.7	18.2	W = 848, p = 0.4915
	Mood	16	64	30.7	28.7	31.4	W = 896, p = 0.1620

## Discussion

Treating epilepsy in adults with ID may be complicated by the presence of a wide range of impairments, the relatively frequent presence of comorbid conditions and by the various residential arrangements used by people with ID. Addressing the challenges associated with these complexities has led to the development of a variety of potential treatment services differing in their skill sets and treatment focus. Thus, the question arises as to whether particular clinical services should be directed towards particular sub-groups of patients.

In the UK most epilepsy management for adults with ID is provided by Neurology or CIDTs or some combination of these services, with varying involvement from primary care professionals. Little research has been undertaken to investigate differences between these settings and services. The aims of this study were to investigate possible differences amongst individuals with ID and epilepsy, between those receiving support from one of these two service alternatives, either in terms of their individual characteristics (described here as casemix variables) or in terms of their outcomes (ELDQoL and GEOS-35) on established instruments.

In considering these issues, we noted that it was not always clear which clinical service was responsible for directing the management of an individual’s epilepsy. We therefore recommend that where more than one clinical service becomes involved in delivering epilepsy-related healthcare to an individual with ID, the relative roles of each are clearly defined and agreed by all concerned. In the small number of study participants for whom the service primarily responsible for managing their epilepsy could not be established there was a relative preponderance of people with severe (5/11) or profound (4/11) ID, a diagnosis of a current neurological comorbidity, and prescription of fewer anti-epileptic drugs. This may indicate that those with the most severe and complex physical comorbidities find it more difficult to access any secondary care services, suggesting that clinicians should pay special attention to how the most severely disabled are treated

Turning to the comparison of characteristics of the participants being supported by CIDT to those being supported by Neurology, some differences were seen in patterns of psychiatric comorbidity and accommodation, but no significant overall differences were found in the profile of ID severity or the number of seizure types reported (Table 2). This suggests that the placement within a particular type of clinical service, at this stage in the participants’ epilepsy career, may not directly relate to their level of ID or their epilepsy. Further, given that differences in ID level were controlled for in the above comparison, the observations that participants whose epilepsy care was managed by a Neurology service were more likely to live in the family home and less likely to have a current comorbid psychiatric diagnosis cannot be explained by these participants having less severe ID. It was also noted that participants under the care of Neurology services were more likely to have seen an Epilepsy Nurse Specialist within the last year: this difference arose as a consequence of the majority of CIDTs included in the study area not having an Epilepsy Nurse.

Considering the comparison of outcome measures describing seizure severity and epilepsy-related quality of life, we examined participants’ scores on the GEOS-35 and ELDQoL. No significant differences were found between participants managed by CIDT and Neurology in terms of these outcome measures. These results can be interpreted to suggest that *either* the choice of clinical service does not affect a service user’s outcome (as measured by these scales), *or*, if the choice of service did originally affect a service user’s outcomes, the differences diminish over time and are no longer evident years later. This may reflect that service users are being supported by the most appropriate clinical service and in both services are being helped as far as current treatments permit. Alternatively, it may reflect that it does not matter which service supports a service user; perhaps, on average, both services eventually achieve a similar level of outcome. However, this comparison of outcomes between the clinical services takes no account of individual patient characteristics (as measured by the casemix variables), which were found to be reasonable predictors of scores (explaining notable proportions of variation: 0.2<R^2^<0.5 –see S1 Table) on the outcome measures. Thus, when comparing outcomes between clinical services, the outcomes would ideally have been adjusted to take account of the individual participant characteristics. However, we have not done this as our sample size would not robustly support such comparisons.

This study has a number of limitations. The work presented here is one part of a larger descriptive body of work investigating epilepsy treatment in those with ID using the recruited cohort: we explore the cost drivers of epilepsy treatment in this population in Pennington et al. [14] whilst Redley et al. [15] looks at the involvement of parents in decision making around epilepsy treatments. As such, there was no single outcome or variable that we were primarily focused and so, with no primary outcome, a sample size calculation was not appropriate. This means that the study was not powered to detect differences between the management pathways on the GEOS-35 and ELDQoL (or other differences). Thus, the lack of statistical differences in these outcomes may be caused by a lack of statistical power. Further, as noted above, we were not able to adjust the outcome comparison for patient characteristics, due to the sample size (small in absolute terms, but from a difficult to recruit vulnerable population).

The study is also limited by having a cross-sectional design, which limits some of the questions we can address. We cannot explore how outcomes fluctuate within the pathways over time. Also, we do not have access to data describing participants’ state at the time of initial referral to the clinical service in which their epilepsy was first managed. Therefore, we cannot comment on the reasons for their referral to one service rather than the other, or comment on earlier aspects of their care pathway experience. To explore outcomes over time and collect robust historical data on the patient treatment would require a participant-demanding longitudinal study in this vulnerable population. However, the cross-sectional study reported here can help focus future research questions, in that we describe individuals, their epilepsy management and related variables in the midst of what for many of the study population has been a very long-term condition, addressing the question of what factors to consider when identifying an appropriate care pathway for individuals with chronic complex comorbidities.

We have not been able to explore the extent of any selection bias. This is potentially concerning, particularly as only 30% (91/306) of eligible patients consented. However, low consent rates are not unusual for research in ID: in Perez et al. [26]–a study looking at adverse outcomes in adults with ID and mealtime support—only 142 consent to take part out of an identified population of 726, a rate of 20%. It is difficult to recruit from the ID population for many reasons, such as the capacity to consent and, relatedly, the frequent need to include family and carers in the research. Unfortunately, we did not have ethical permission to retain the data of non-responders or of the non-consenting population and so cannot conduct a non-responder analysis to compare the characteristics of those that did and did not consent. However, we can compare some of our consenter characteristics with a meta-analysis conducted by Robertson et al. [27] that considers 48 studies of epilepsy in people with ID. We found that 15% of our sample had mild ID, while the remaining 85% had more severe ID; this compares with a split of 17.5% and 82.5% within the meta-analysis of Robertson et al [27]. Further, the meta-analysis shows the prevalence of epilepsy within people with ID to be similar in both males (24.8%, 95% CI: 19.6–30.8%) and females (22.2%, 95% CI: 17.3–28.1); therefore in our sample, all of whom have ID and epilepsy, we would expect a near equal split between the sexes—this is observed (51% male/49% female). Hence, for at least these parameters, there is evidence for our population being representative of the wider population with ID and epilepsy.

In summary, this research has demonstrated that, when looked at cross-sectionally, there is no difference in seizure severity and epilepsy-related quality of life between those adults with ID and epilepsy whose seizures are managed by a Neurology service and those whose seizures are managed by a CIDT. With respect to the question of why participants were receiving care from one type of service rather than another, the original reason for referral was not investigated in this study. However, it is noted that there were differing rates of comorbidity, with those in Neurology services having more neurological comorbidities whilst those in CIDTs had more psychiatric comorbidity. The question as to whether this pattern reflects reasons behind the original choice of referral route or perhaps suggests that Neurologists identify additional neurological diagnoses whilst patients in CIDTs, staffed by mental health and social care staff, are more likely to be diagnosed with psychopathology, remains to be established.

## Supporting information

S1 Table The structure of final linear models relating casemix variables to the outcome scales. (DOCX)
