# Supplementary material for: Clinical services for adults with an intellectual disability and epilepsy: A comparison of management alternatives
Source: PLoS One. 2017 Jul 3;12(7):e0180266. doi: 10.1371/journal.pone.0180266 (PMC5495336; doi:10.1371/journal.pone.0180266)
Supplement: S1 Table — (DOCX) [file pone.0180266.s001.docx]

**Supplementary 1 Table.** **The structure of final linear models relating casemix variables to the outcome scales.**

| **Linear model fit to** | |  | **Variables remaining after AIC backwards elimination** | | | | | | |  |  |
| --- | --- | --- | --- | --- | --- | --- | --- | --- | --- | --- | --- |
|  |  | **N** | **ID severity** | **Accommodation** | **#AEDs (0-1 or 2+)** | **Seen ENS** | **Current comorbid neurological diagnosis** | **Current comorbid psychiatric diagnosis** | **(Max) Seizure types reported** | **R^2^** | **Relationships - higher scores from those who:** |
| **GEOS-35** | **Seizures** | 85 | √ |  |  |  |  |  | √ | .41 | - Have severe ID - Report more seizure types |
|  | **Medical treatment^a^** | 85 | √ |  | √ |  |  |  | √ | .35 | - Have severe ID - Take 2+ AEDs - Report more seizure types |
|  | **Caring** | 84 | √ | √ | √ | √ |  |  | √ | .43 | - Have severe ID - Live with family - Take 2+ AEDs - Have seen an ENS - Report more seizure types |
|  | **Social impact^b^** | 82 |  |  |  | √ |  |  | √ | .18 | - Have seen an ENS - Report more seizure types |
|  | **Total^c^** | 85 | √ |  | √ | √ |  |  | √ | .46 | - Have severe ID - Take 2+ AEDs - Have seen an ENS - Report more seizure types |
| **ELDQoL** | **Seizure severity** | 86 |  |  | √ |  |  |  | √ | .18 | - Take 2+ AEDs - Report 1 or 3+ seizure types |
|  | **Side effects** | 84 | √ |  | √ |  |  |  | √ | .34 | - Have severe ID - Take 2+ AEDs - Report more seizure types |
|  | **Behaviour** | 86 | √ |  |  |  | √ |  | √ | .33 | - Have severe or  profound ID - Have a current comorbid neurological diagnosis - Report more seizure types |
|  | **Mood** | 85 |  | √ | √ |  |  | √ | √ | .33 | - Live in group homes/ supported living - Have 2+ AEDs - Have a current comorbid psychiatric diagnosis - Report 3+ seizure types |

GEOS-35 = Glasgow Epilepsy Outcome Scales-35. ELDQoL = Epilepsy and Learning Disabilities Quality of Life. ID = Intellectual Disability. AEDs = Anti-Epileptic Drugs. ENS = Epilepsy Nurse Specialist.

a = Response is (Medical treatment)^0.4.

b = Response is log(Social impact).

c = Response is (Total)^0.3.
